# Uncovering Distinct Primary Vaccination-Dependent Profiles in Human *Bordetella pertussis* Specific CD4+ T-Cell Responses Using a Novel Whole Blood Assay

**DOI:** 10.3390/vaccines8020225

**Published:** 2020-05-15

**Authors:** Eleonora E. Lambert, Véronique Corbière, Jacqueline A. M. van Gaans-van den Brink, Maxime Duijst, Prashanna Balaji Venkatasubramanian, Elles Simonetti, Martijn Huynen, Dimitri D. Diavatopoulos, Pauline Versteegen, Guy A. M. Berbers, Françoise Mascart, Cécile A. C. M. van Els

**Affiliations:** 1 Center for Infectious Disease Control, National Institute for Public Health and the Environment (RIVM), 3721 MA Bilthoven, The Netherlands; nora.lambert@rivm.nl (E.E.L.); jacqueline.van.gaans@rivm.nl (J.A.M.v.G.-v.d.B.); duijstmaxime@gmail.com (M.D.); pauline.versteegen@rivm.nl (P.V.); guy.berbers@rivm.nl (G.A.M.B.); 2 Laboratory of Vaccinology and Mucosal Immunity, Université Libre de Bruxelles (U.L.B.), 1070 Brussels, Belgium; veronique.corbiere.ulb@gmail.com (V.C.); francoise.mascart@erasme.ulb.ac.be (F.M.); 3 Center for Molecular and Biomolecular Informatics, Radboud Institute for Molecular Life Sciences, Radboudumc, 6525 GA Nijmegen, The Netherlands; balaji.venkatasubramanian@radboudumc.nl (P.B.V.); martijn.huijnen@radboudumc.nl (M.H.); 4 Laboratory of Medical Immunology, Radboud Institute for Molecular Life Sciences, Radboudumc, 6525 GA Nijmegen, The Netherlands; elles.simonetti@radboudumc.nl (E.S.); dimitri.diavatopoulos@radboudumc.nl (D.D.D.)

**Keywords:** *Bordetella pertussis*, whole blood assay, CD4+ T-cells, Th polarization, Th cytokines, booster vaccination, vaccination background

## Abstract

To advance research and development of improved pertussis vaccines, new immunoassays are needed to qualify the outcome of *Bordetella pertussis* (Bp) specific CD4+ T-cell differentiation. Here, we applied a recently developed whole blood assay to evaluate Bp specific CD4+ T-cell responses. The assay is based on intracellular cytokine detection after overnight *in vitro* Bp antigen stimulation of diluted whole blood. We show for the first time that CD4+ T-cell memory of Th1, Th2, and Th17 lineages can be identified simultaneously in whole blood. Participants ranging from 7 to 70 years of age with different priming backgrounds of whole-cell pertussis (wP) and acellular pertussis (aP) vaccination were analyzed around an acellular booster vaccination. The assay allowed detection of low frequent antigen-specific CD4+ T-cells and revealed significantly elevated numbers of activated and cytokine-producing CD4+ T-cells, with a significant tendency to segregate recall responses based on primary vaccination background. A stronger Th2 response hallmarked an aP primed cohort compared to a wP primed cohort. In conclusion, analysis of Bp specific CD4+ T-cell responses in whole blood showed separation based on vaccination background and provides a promising tool to assess the quantity and quality of CD4+ T-cell responses induced by vaccine candidates.

## 1. Introduction

Pertussis, a highly infectious respiratory illness caused by the human-restricted pathogen *Bordetella pertussis* (Bp), is endemic despite global vaccination. The first whole-cell pertussis (wP) vaccines were introduced in the 1940s/1950s and were based on formalin-inactivated bacteria representing a plenitude of bacterial components and innate ligands. This composition provided the induction of broad protective immunity against disease, however also caused side effects such as fever and inflammation [1,2,3]. In many countries, wP vaccines were replaced by the safer acellular pertussis (aP) vaccines two to three decades ago. aP vaccines contain one to five of the major immunogenic virulence factors: pertussis toxin (PT), in combination with filamentous hemagglutinin (FHA), pertactin (Prn), and fimbriae 2/3, and are aluminum adjuvanted. Epidemiological data indicate that duration of protection is shorter after aP vaccines compared to wP vaccines or natural infection [4,5,6,7]. Characterization of the induced immune responses indicated essential differences in functional polarization of Bp specific CD4+ T-cells by wP and aP vaccines [8,9,10,11], which were found to be maintained into adolescence and adulthood [10,11,12,13,14,15] and could relate to the epidemiological observations on duration of protection. Priming of infants with an aP vaccine induces mixed polarized CD4+ T-cell immunity which is Th2 skewed, while wP primed CD4+ T-cell immunity in animal models is Th1/Th17 polarized which is comparable to what is found after natural infection [16,17,18]. This opposite functional priming of Bp specific CD4+ T-cell responses is corroborated by studies in animal models, where it has been shown that Th1/Th17 type immunity is required for protection against a bacterial challenge [19,20,21,22,23]. Suboptimal induction of cellular immune responses by the current aP vaccines indicate the need for an effective, safe third generation of vaccines that can induce a durable and protective type of CD4+ T-cell memory. New (candidate) vaccines will have to be evaluated in field trials based on the induction of correlates of protection [24]. This will need new assays that can monitor Bp specific CD4+ T-cells and simultaneously assess to which Th lineage they belong, preferably in a rapid, real-time, and blood saving format. Classical assays using peripheral blood mononuclear cells (PBMCs) require a relatively large blood volume and cannot exclude activation of lymphocyte subsets during the isolation procedure. For other pathogens, such as *Mycobacterium tuberculosis*, *Mycobacterium leprae*, cytomegalovirus, and dengue virus, whole blood assays have been used to detect cell-mediated immunity to infection or vaccination [25,26,27,28,29]. These are based on short in vitro stimulation with specific antigens and have intracellular cytokine staining of T-cells as a main readout.

Within the framework of the European PERISCOPE project/consortium, we for the first time developed a flow cytometric whole blood assay allowing simultaneous detection of Bp specific Th1, Th2 and Th17 type cytokine producing CD4+ T-cells as main readout. Here, this assay, based on short *in vitro* stimulation with Bp specific antigens, was used in a large ongoing clinical booster vaccination study in children, adolescents, and young and older adults with different primary vaccination backgrounds. The dynamics of Bp specific CD4+ T-cell responses of all Th lineages and the imprinting effects of primary vaccination in the different cohorts were assessed.

## 2. Materials and Methods

### 2.1. Ethical Statement

Participants donating whole blood were healthy Dutch participants included in a clinical study (acronym: BERT study), which is described in detail elsewhere (Versteegen et al., in preparation). The clinical study was registered at the European Clinical Trials register under the study number: 2016-003678-42 and approved by the accredited Medical Research Ethics Committee Utrecht. All participants and parents/guardians of minor participants provided written informed consent. This study was conducted in compliance with the principles of the Declaration of Helsinki.

### 2.2. Study Population and Booster Vaccination

For *ex vivo* T-cell analysis, 73 healthy participants were included. *n* = 19 were 7–10 years of age (referred to as children) with an aP priming background, *n* = 24 were 11–15 years of age (adolescents) with either an aP or a wP priming background, *n* = 15 were 20–34 years of age (young adults) with a wP priming background and *n* = 15 were 60–70 years of age (older adults) with a unknown proportion of wP priming background or no vaccination background. The distribution of male and female participants and age is indicated in Table 1. All participants received one dose of an aP vaccine included in a combination vaccine (Tdap)-IPV (Boostrix^®^-IPV, GlaxoSmithKline, Wavre, Belgium).

### 2.3. Whole Blood Sampling

During home visits, blood was drawn by venipuncture at baseline (day 0), after which the booster vaccination was administered to participants. At subsequent home visits, depending on the age cohorts, at day 14 and day 28 post booster vaccination, blood was drawn. Blood was collected in sodium heparin tubes (Vacuette, Greiner Bio-One, Kremsmünster, Austria), transported to the laboratory (National Institute for Public Health and the Environment, Bilthoven, The Netherlands) and used in real-time assays within 12 h after collection (median time between blood collection and culture was 6 h, with an interquartile range of 4.3–8.5 h).

### 2.4. Antigens, Peptides and Co-Stimulants

Purified genetically-detoxified Pertussis Toxin (PT; R9K, E129A; Limulus Amebocyte Lysate activity < 20 EU/mg) was purchased from LIST Biological laboratories Inc. (Campbell, CA, USA) and stored at 500 µg/mL at −20 °C. Purified Filamentous hemagglutinin (FHA) was kindly provided by Sanofi Pasteur (Marcy-l’Étoile, France) and stored at 700 µg/mL at 4 °C (Limulus Amebocyte Lysate activity < 66 EU/mg). A bacterial lysate of Bp strain B1917 [30] (Bp lysate/BPL) was kindly provided by Q Biologicals (Ghent, Belgium) and stored at 3.4 mg/mL at −80 °C. A peptide pool of 132 Bp immunogenic peptide sequences (referred to as peptide pool) from the five Bp vaccine antigens [15] was purchased from Pepscan (Lelystad, the Netherlands) and stored as a mix at 0.1 mM per peptide in PBS/10% DMSO at −20 °C. *Staphylococcus aureus* enterotoxin B (SEB) was purchased from Sigma (Saint Louis, MO, USA) and stored at 1 mg/mL at −20 °C. Purified anti-CD28 antibodies (stock concentration 1 mg/mL) and anti-CD49d antibodies (stock concentration 1 mg/mL) were purchased from eBiosciences (Landsmeer, The Netherlands). PT and Bp lysate were heat-inactivated for 10 min at 80 °C using a water bath to avoid any mitogenicity in the whole blood stimulations.

### 2.5. PerfectCount and CD4+ Euroflow T-Cell Tubes

To enumerate absolute CD4+ T-cell events per μL of whole blood samples, 50 and 100 μL aliquots of freshly drawn whole blood were assessed in a PerfectCount tube and a CD4+ Euroflow T-cell tube, respectively, as described earlier [31].

### 2.6. Whole Blood Stimulation

Fresh whole blood samples were aliquoted (400 μL per condition), diluted 1:1 with RPMI 1640 medium supplemented with GlutaMAX (Gibco, Leiden, The Netherlands) with 5% Penicillin/Streptomycin (Invitrogen, Carlsbad, CA, USA) and incubated for 19 h at 37 °C, 5% CO_2_, 100% humidity in round bottom 15 mL tubes, loosely covered by caps. Different parallel stimulation conditions were performed, i.e., incubation in the presence of heat-inactivated PT (5 μg/mL), FHA (5 μg/mL), heat-inactivated Bp lysate (10 μg/mL), peptide pool (at 0.1 µM), SEB (1 μg/mL) or in the absence of antigen (hereafter: unstimulated). Except for SEB, all stimulation conditions (including the unstimulated controls) were cultured in the presence of co-stimulatory anti-CD28 and anti-CD49d antibodies both at a final concentration of 1 μg/mL. Cellular protein transport was inhibited for the last 5 h of incubation to capture intracellular cytokines, by adding to the tubes GolgiStop (BD Biosciences, San Jose, CA, USA) and GolgiPlug (Sigma, St. Louis, MO, USA), both at a final concentration of 1 µg/mL.

### 2.7. Processing Whole Blood

After 19 h incubation with antigens, the samples were pelleted, and culture supernatants were harvested. Erythrocyte lysis was performed on the pelleted cells (PharmLyse, BD Biosciences) for 10 min at room temperature (RT) in the dark, and subsequently the cell pellet was fixed for 20 min at RT in the dark and permeabilized for 10 min at RT in the dark according the kit protocol (Cytofix/Cytoperm Solution kit for whole blood, BD Biosciences).

### 2.8. Staining of the Cells for Flow Cytometry and Acquisition of the Data

After permeabilization, surface and intracellular staining of cells was combined. The antibody panel included CD3-APC-H7 (BD Biosciences, clone SK7), CD4-BB515 (BD Biosciences, clone SK3), CD8-PerCP-Cy5.5 (BD Biosciences, clone SK1), CD154-APC (BD Biosciences, clone TRAP-1), IFNγ-BV510 (BD Biosciences, clone B27), IL-5-BV421 (BioLegend, San Diego, CA, USA; clone TRFK5), IL-13-BV421 (BD Biosciences, clone JES10-5A2), IL-17A-PE (BioLegend, clone BL168), IL-17F-PE (BD Biosciences, clone 033-782), and IL-22-PE-Cy7 (eBiosciences, clone 22 URTI) in Perm/wash buffer (Cytofix/Cytoperm kit, BD Biosciences), for 30 min at RT in the dark. Cells were washed, recovered in buffer (PBS with 0.1% NaAz and 0.5% BSA) and completely acquired on a FACS Canto II (BD Biosciences). Every experimental day, instrument performance was checked with 8-peaks Rainbow Beads to assure MFIs were in range.

### 2.9. Multiplex Cytokine Assay

A commercial LEGENDplex Human Th Cytokine panel 13-plex (BioLegend, San Diego, CA, USA) was used to determine concentrations of IL-2, IL-4, IL-5, IL-6, IL-9, IL-10, IL-13, IL-17A, IL-17F, IL-21, IL-22, IFNγ, and TNF in the culture supernatants of stimulated blood samples.

### 2.10. Data Analysis

All flow cytometric data were analyzed using FlowJo software, (version 10, TreeStar, Inc., Ashland, OR, USA). Statistical analyses were done with GraphPad Prism (version 8.2.1, San Diego, CA, USA) and data were presented as medians or geomeans with quartiles or 5–95 percentiles, as indicated. Culture supernatant data were analyzed using LEGENDplex software, version 8.0 (BioLegend, San Diego, CA, USA). Non-parametric statistical tests were used, namely the Wilcoxon signed-rank test for analysis of paired data, Kruskal–Wallis test or the mixed-effect analysis followed by multiple comparisons corrected with Dunnett’s test. Statistical programming language, R, was used for PCA, correlation analysis and for predictive modelling using elastic net [32]. Correlation plots were made on the culture supernatant data after subtracting values from unstimulated control supernatants data using “corrplot” package while elastic net was performed using “glmnet” package in R [33,34]. The elastic net model was applied on the culture supernatant data after subtracting values from unstimulated control supernatants, followed by a log transformation of the raw data. All negative values in the untransformed data were reset as 0.01. The model was tuned to search for the optimal regularization parameters using internal cross validation and was iterated 1000 times to identify the most relevant antigen-cytokine pairs. In each iteration, the data was randomly split into a training set (70%) and a test set (30%) and models with a Receiver Operating Characteristic (ROC) greater than 0.93 were chosen for parameter selection [32].

## 3. Results

### 3.1. Study Design to Evaluate Bp Specific CD4+ T-Cell Immunity in a Whole Blood Assay

Whole blood samples, collected longitudinally from 73 participants representing four age cohorts in a clinical study (Table 1), were used to evaluate Bp specific CD4+ T-cell responses around an acellular pertussis booster vaccination with a registered combination vaccine. Cohorts represented different age strata and primary pertussis vaccination backgrounds, i.e., children (7–10 years of age, aP primed), adolescents (11–15 years of age, 46% aP, 54% wP primed), young adults (20–34 years of age, wP primed), and older adults (60–70 years of age, wP primed or pre-vaccination era), respectively. In total, 202 whole blood samples were real-time evaluated in a novel Bp specific whole blood T-cell assay developed in the framework of PERISCOPE [24]. According to the protocol, whole blood samples were aliquoted and distributed over 4–6 antigenic stimulation conditions per sample, representing besides unstimulated and positive controls the Bp antigens PT, FHA, Bp lysate, and a Bp peptide pool, for overnight culture until harvested for a primary cell-based and secondary supernatant-based readouts. In total, 941 stimulated cultures from the 202 whole blood samples were generated and evaluated in this study.

### 3.2. CD4+ T-Cell Numbers in Cultured Whole Blood Samples Are Adequate for a Reliable Analysis of Rare Events in All Age Cohorts

**Figure 1:**
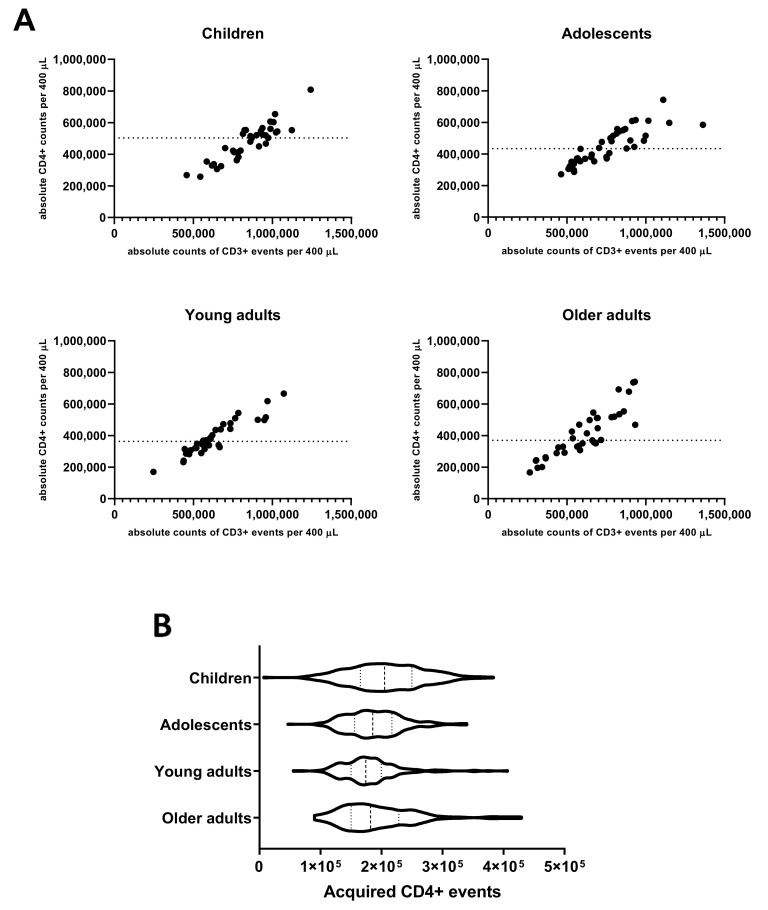
Absolute counts of CD4+ T-cells in 400 µL whole blood before and after culture in different age cohorts. (**A**) Absolute counts of CD3+ and CD4+ events were determined in 100 µL whole blood at day 0 pre-booster, day 14 and day 28 post-booster before culture and extrapolated to the equivalent of 400 µL in children, adolescents, young, and older adults as indicated. Dashed lines indicate median value of absolute CD4+ events per cohort. (**B**) Violin plots showing the variation, quartiles (thin dashed lines), and median values (thick dashed line) of total acquired CD4+ T-cell events from all stimulated cultures in the whole blood assay of all cohorts, as indicated.

As the evaluation of the Bp specific CD4+ T-cell responses in the applied method comprised rare event analysis in cell pellets derived from cultured aliquots of 400 µL whole blood, we determined the absolute input of CD4+ T-cells in this volume based on parallel PerfectCount and TCD4 Euroflow tube testing. The median absolute number of CD3+CD4+ events per 400 µL whole blood found in children (502,902) was higher than in young adults (360,740, *p* = 0.004) and older adults (370,855, *p* = 0.047) and comparable to adolescents (433,673, n.s.) (Figure 1A). Additionally, after stimulation, processing, and complete flow cytometric acquisition of samples, absolute median counts of CD3+CD4+ T-cells per 400 µL cultured whole blood were highest in children (205,222) and significantly lower median counts were found in adolescents (185,675, *p* = 0.0005), young adults (174,362, *p* < 0.0001), and older adults (182,150, *p* = 0.0019), respectively (Figure 1B).

These data indicate that a cohort-dependent yield of 41%, 43%, 47%, and 49% of input CD4+ T-cells was retrieved, respectively, and could be assessed in the assay for CD4+ T-cell responses. Additionally, the results show that with an input of 400 µL of whole blood per stimulation at least 100,000 CD4+ T-cells were acquired after culture by flow cytometry in ≥95% of samples from all age cohorts enabling reliable interrogation of low-frequent subpopulations. To study specific cells in the CD4+ T-cell population with frequencies in the order of 0.02%, it is recommended to acquire at least this number of CD4+ T-cells and to obtain a minimum of 20 events in the end gate [35].

### 3.3. Detection and Monitoring of Bp Specific CD4+ T-Cell Responses Based on CD154 Expression

**Figure 2:**
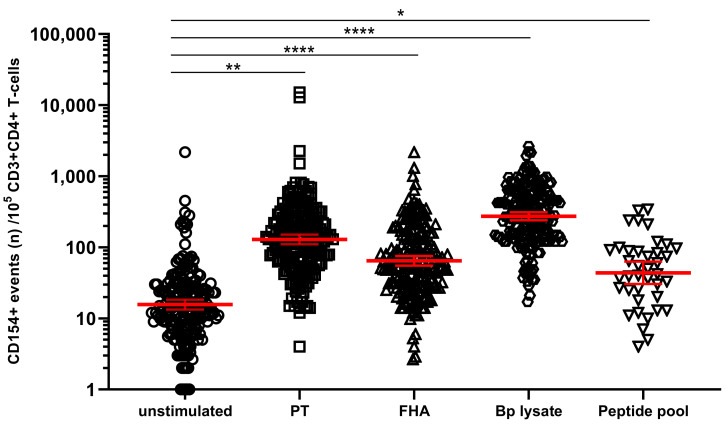
Stimulation with *Bordetella pertussis* (Bp) antigens in vitro increases the number of CD154+ CD4+ T-cells. On days 0, 14, and 28, whole blood samples from all participants in all age cohorts were stimulated with PT (□), FHA (Δ), Bp lysate (⬡), peptide pool (∇) or left unstimulated (ο), and CD154+ events were determined in CD3+CD4+ T-cells. CD154 positive events were expressed per 10^5^ CD3+CD4+ T-cells. Bars (red) indicate geomeans + 95% CI of combined raw data from these time points from all participants in all age cohorts. * *p* < 0.05, ** *p* < 0.01, **** *p* < 0.0001.

In the primary cell-based readout of the assay, identification of CD4+ T-cells specific for Bp is based on detection of upregulated biomarkers only if cultured in the presence of Bp antigen but not in its absence [36,37]. We explored whether CD4+ T-cells in Bp antigen-stimulated conditions had higher expression of activation marker CD154 (CD40L) compared to unstimulated controls (cultured in the absence of antigenic stimulation). The geomean of CD154+ cells per 10^5^ CD3+CD4+ T-cells, in blood samples from all timepoints and age cohorts together, was significantly higher after a short in vitro stimulation with each of the Bp antigens, i.e., 118.1 for PT, 64.4 for FHA, 267.1 for Bp lysate, and 43.1 for peptide pool compared to 14.7 for the unstimulated control (Figure 2).

**Figure 3:**
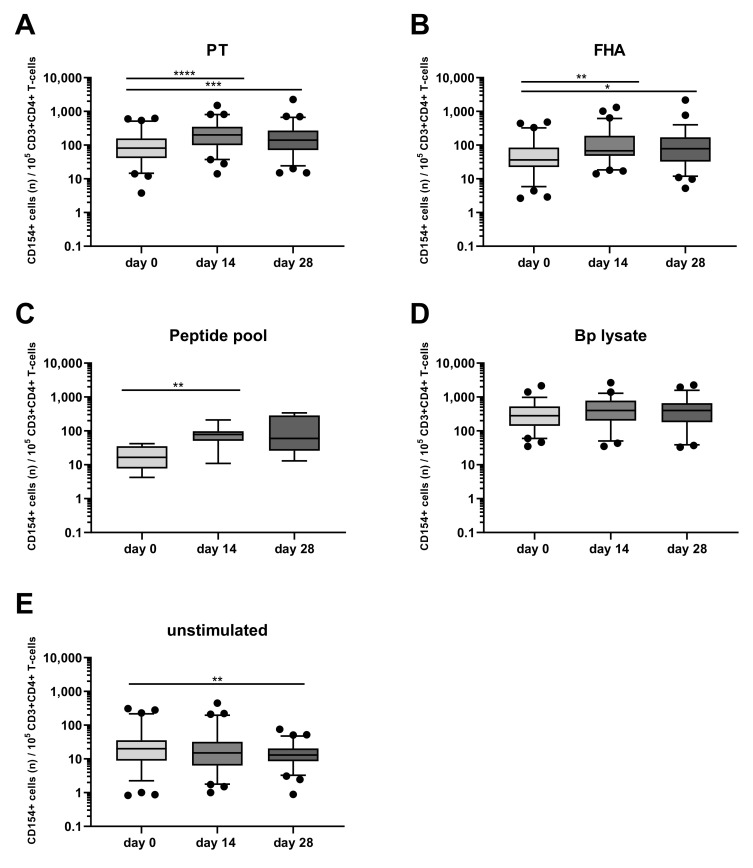
Post-booster vaccination CD4+ T-cell responses are revealed by Bp antigen stimulation and CD154 marker expression. CD154+ events per 10^5^ CD3+CD4+ T-cells were determined in whole blood samples from all four age cohorts, at different timepoints and after stimulation with (**A**) pertussis toxin (PT) [5 µg/mL], (**B**) filamentous hemagglutinin (FHA) [5 µg/mL], (**C**) Bp specific peptide pool [0.1 µM], (**D**) Bp lysate [10 µg/mL], (**E**) unstimulated control, as indicated, all in presence of costimulatory antibodies. Boxplots indicate median value, quartile range, and 5–95 percentiles of raw data. Circles indicate data points outside the 5-95 percentiles. * *p* < 0.05, ** *p* < 0.01, *** *p* < 0.001, **** *p* < 0.0001.

Longitudinal analysis around the administration of the vaccine booster dose indicated a significant increase of PT- and FHA-stimulated CD154+CD4+ T-cells both at days 14 and 28 (with typical median levels of 200 and 68 activated cells per 10^5^ CD4+ T-cells for PT and FHA at day 14, respectively) compared to the results obtained at day 0 pre-booster (Figure 3A,B), whereas the peptide pool only revealed significant activation of cells at day 14 (Figure 3C). An overall high level of CD154+CD4+ T-cells after Bp lysate stimulation was found (Figure 3D), with no change over time compared to pre-booster blood. This can be interpreted as a consequence of the crudeness of the Bp lysate containing Bp specific as well as common bacterial antigens and innate receptor triggering ligands that may induce background activation of CD4+ T-cells. In contrast, the rate of activated CD4+ T-cells detected in unstimulated blood was generally low before and after the booster, lowest at day 28 (median 13 CD154+ events per 10^5^ CD4+ T-cells (Figure 3E). The positive control on the other hand, comprised high amounts of CD154+CD4+ T-cells that remained unchanged over time as expected (Figure S1). These data indicate that the novel whole blood flow cytometric assay was capable of detecting responses of low frequent Bp specific CD4+ T-cells upon booster vaccination as shown by their upregulated expression of CD154 after in vitro PT, FHA, and peptide pool but not Bp lysate stimulation, and that activated Bp specific CD4+ T-cells are enhanced at days 14 and 28 after booster vaccination.

### 3.4. Detection of Bp Specific CD4+ T-Cells by Their Intracellular Cytokine Profile

**Figure 4:**
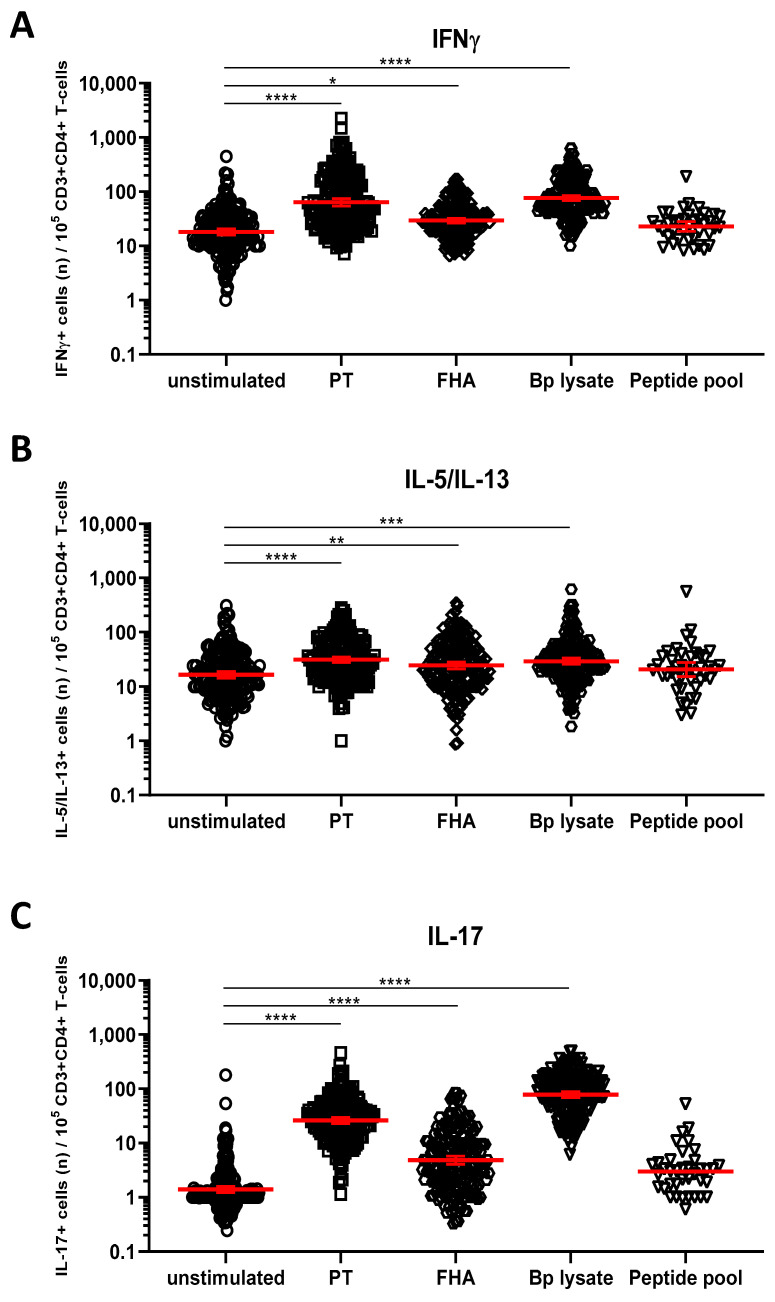

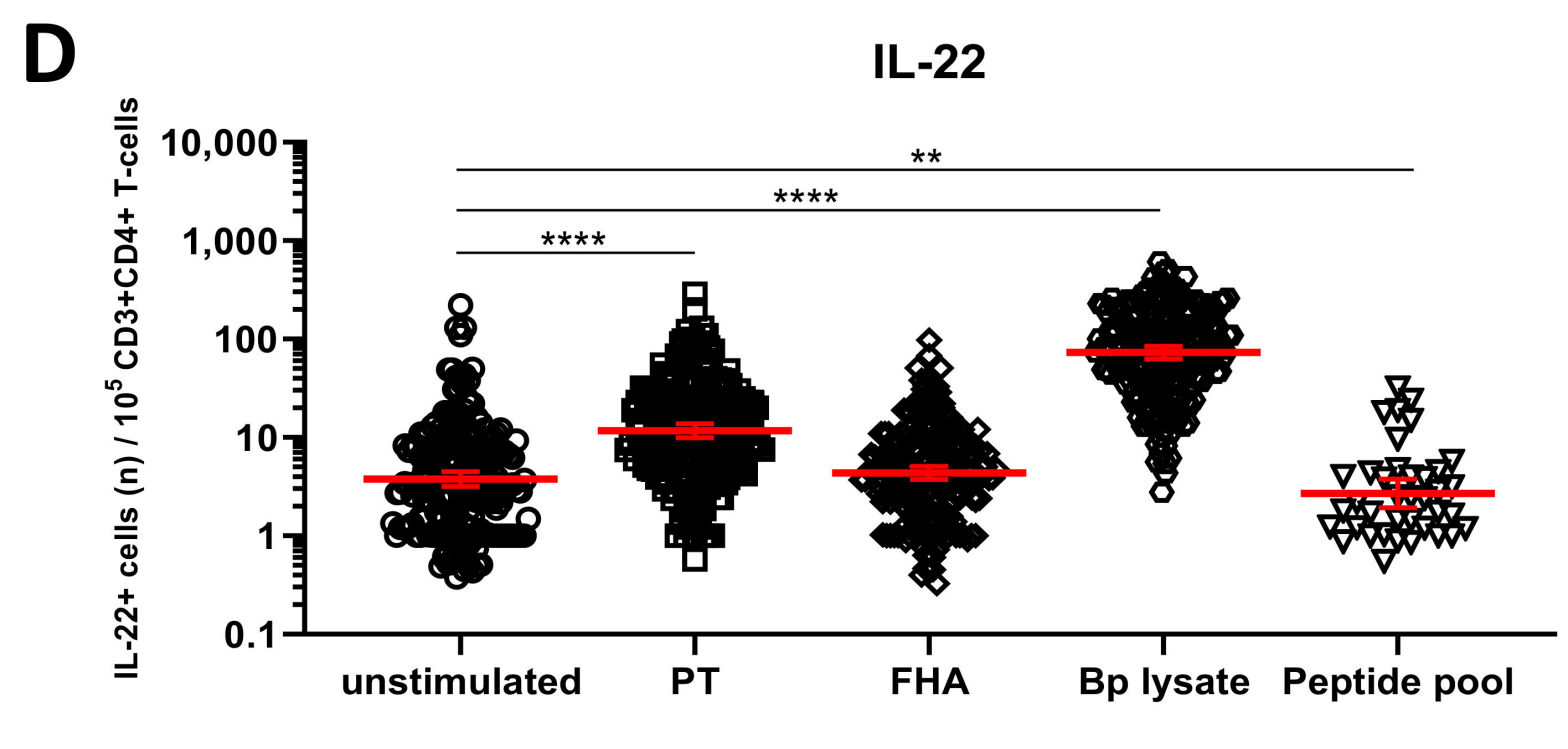
Detection of intracellular cytokines in CD4+ T-cells after Bp antigen stimulation. On days 0, 14, and 28, whole blood samples from all participants in all age cohorts were stimulated with PT (□), FHA (Δ), Bp lysate (⬡), peptide pool (∇) or left unstimulated (ο), as indicated, and intracellularly stained for signature cytokines of Th1, Th2, and Th17 responding cells. Cytokine positive events were expressed per 10^5^ CD3+CD4+ T-cells for (**A**) IFNγ, (**B**) IL-5/IL-13, (**C**) IL-17A/IL-17F, (**D**) IL-22. Bars (red) indicate geomeans +95% CI of combined raw data from all time points from all participants in all age cohorts. * *p* < 0.05, ** *p* < 0.01, *** *p* < 0.001, **** *p* < 0.0001.

We next explored whether short in vitro stimulation with Bp antigens could reveal the presence and patterns of Bp specific Th1, Th17, and Th2 cells. IFNγ+CD4+ T-cells were taken as a readout for Th1 cells, whereas IL-17A/IL-17F+CD4+ (hereafter IL-17+CD4+) and IL-5/IL-13+CD4+ T-cells represented Th17 and Th2 cells, respectively. IL-22+CD4+ T-cells were analyzed as well, as IL-22 is a cytokine also produced by Th17 cells. Combined results obtained for all samples from the different cohorts indicated significantly increased geomean numbers of IFNγ+ cells per 10^5^ CD4+ T-cells after PT (64, *p* < 0.0001), FHA (29, *p* = 0.0108), and Bp lysate (78, *p* < 0.0001) but not peptide pool (24, ns.) (Table S2) stimulation compared to the unstimulated control (19) (Figure 4A, Table S1). Similarly, significant increases in geomean numbers were observed for IL-5/IL-13+ cells per 10^5^ CD4+ T-cells after PT (31, *p* < 0.0001), FHA (24, *p* = 0.0016), and Bp lysate (30, *p* < 0.0001) but not peptide pool (21, ns.) stimulation, compared to unstimulated (17) (Figure 4B, Table S1), as well as for IL-17+ cells per 10^5^ CD4+ T-cells after PT (25, *p* < 0.0001), FHA (5, *p* < 0.0001), and Bp lysate (78, *p* < 0.0001) but not peptide pool (3, ns.) stimulation, compared to unstimulated controls (1) (Figure 4C, Table S1). Notably, rates of IL-22+ cells in CD4+ T-cells were increased in response to PT (12, *p* < 0.0001) and Bp lysate stimulation (75, *p* < 0.0001) as compared to unstimulated controls (4) but not with the peptide pool (3, *p* = 0.0016) (Figure 4D, Table S1).

### 3.5. Kinetics of the Bp Specific CD4+ T-Cells after an aP Booster Vaccination Per Age Cohort

The kinetics of the Bp specific CD4+ T-cells after the booster dose were further analyzed in the different age cohorts, hereby focusing on the responses induced by Bp antigens PT and FHA, which revealed specific booster responses by activation marker expression as well as significant intracellular cytokine responses, as demonstrated in the previous sections.

**Figure 5:**
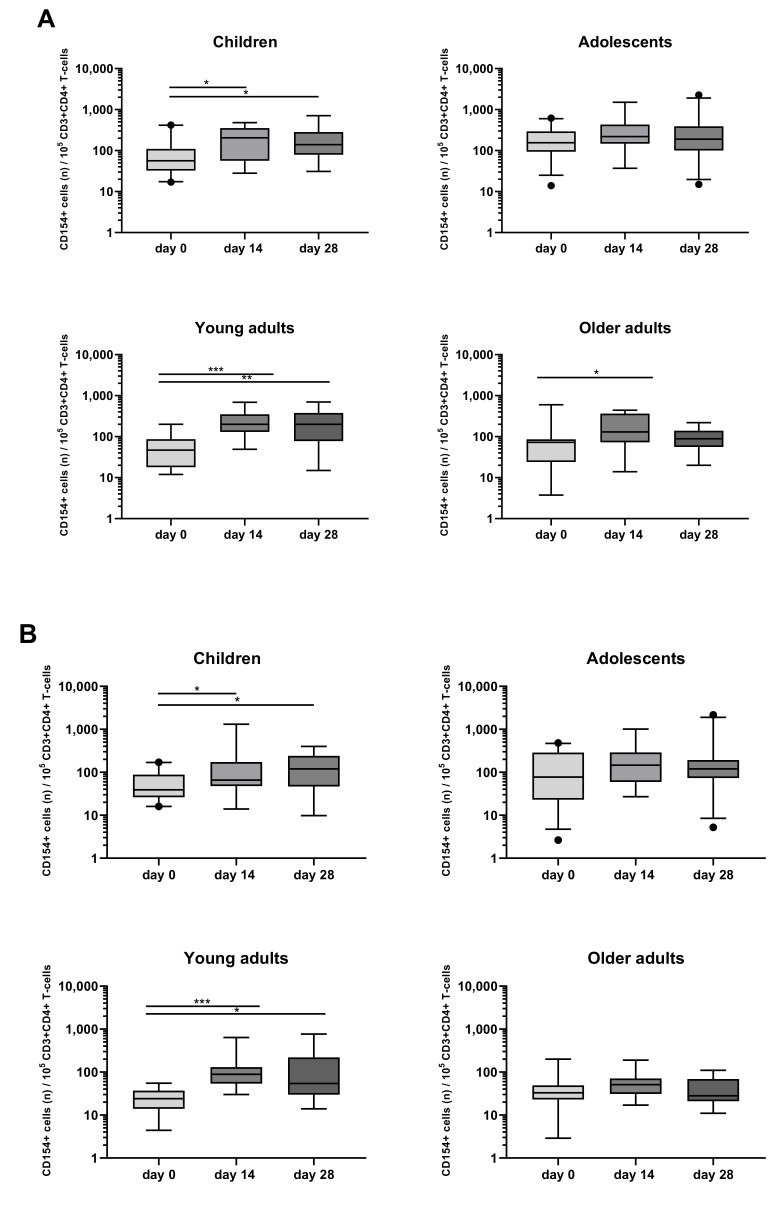
Longitudinal CD154 expression of CD4+ T-cells after PT and FHA stimulation in the different age cohorts. CD154+ events per 10^5^ CD3+CD4+ T-cells were determined in whole blood samples of the four separate age cohorts, at different timepoints and after stimulation with (**A**) PT, (**B**) FHA, in the presence of costimulatory antibodies. Boxplots indicate median value, quartile range, and 5–95 percentiles of raw data. Circles indicate data points outside the 5-95 percentiles. * *p* < 0.05, ** *p* < 0.01, *** *p* < 0.001.

When looking at the activation marker, a significant increase in the rate of CD154+CD4+ T-cells after PT stimulation was found in children (day 14, day 28), young adults (day 14, day 28), and older adults (day 14) (Figure 5A). A significant increase of CD154+CD4+ T-cells was also found after FHA stimulation in children (day 14, day 28) and young adults (day 14, day 28) (Figure 5B).

**Figure 6:**
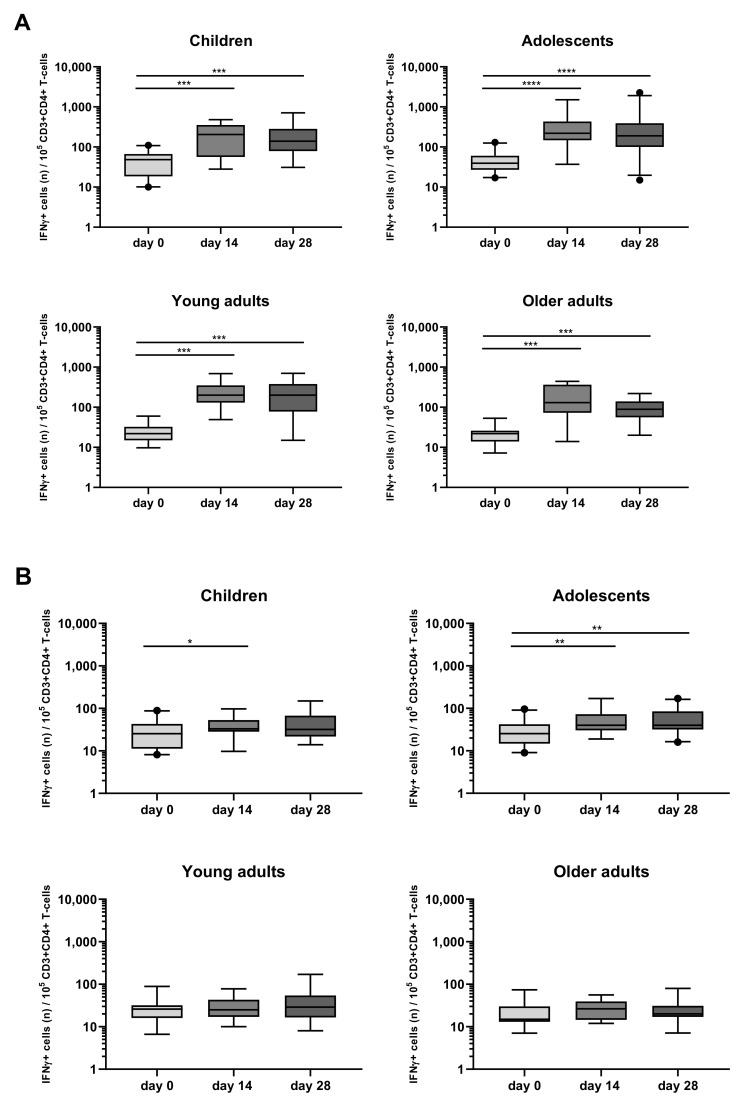
Longitudinal IFNγ responses in CD4+ T-cells after PT and FHA stimulation in the different age cohorts. Whole blood samples were stimulated with antigens (**A**) PT and (**B**) FHA, and longitudinal IFNγ responses were determined after culture, by intracellular cytokine staining in children, adolescents, young, and older adults, as indicated. Responses are expressed as positive events per 10^5^ CD3+CD4+ T-cells. Boxplots indicate median value, quartile range, and 5–95 percentiles. Circles indicate data points outside the 5-95 percentiles. * *p* < 0.05, ** *p* < 0.01, *** *p* < 0.001, **** *p* < 0.0001.

When looking at intracellular cytokine expression, a significant increase in the rate of IFNγ+CD4+ T-cells in response to PT at days 14 and 28 was found as compared to day 0 in all four different cohorts (Figure 6A, Table S2). In contrast, FHA stimulation associated less frequently and less significantly with IFNγ+CD4+ T-cell responses which were noticed only at day 14 in children and in adolescents at days 14 and 28 (Figure 6B, Table S2).

**Figure 7:**
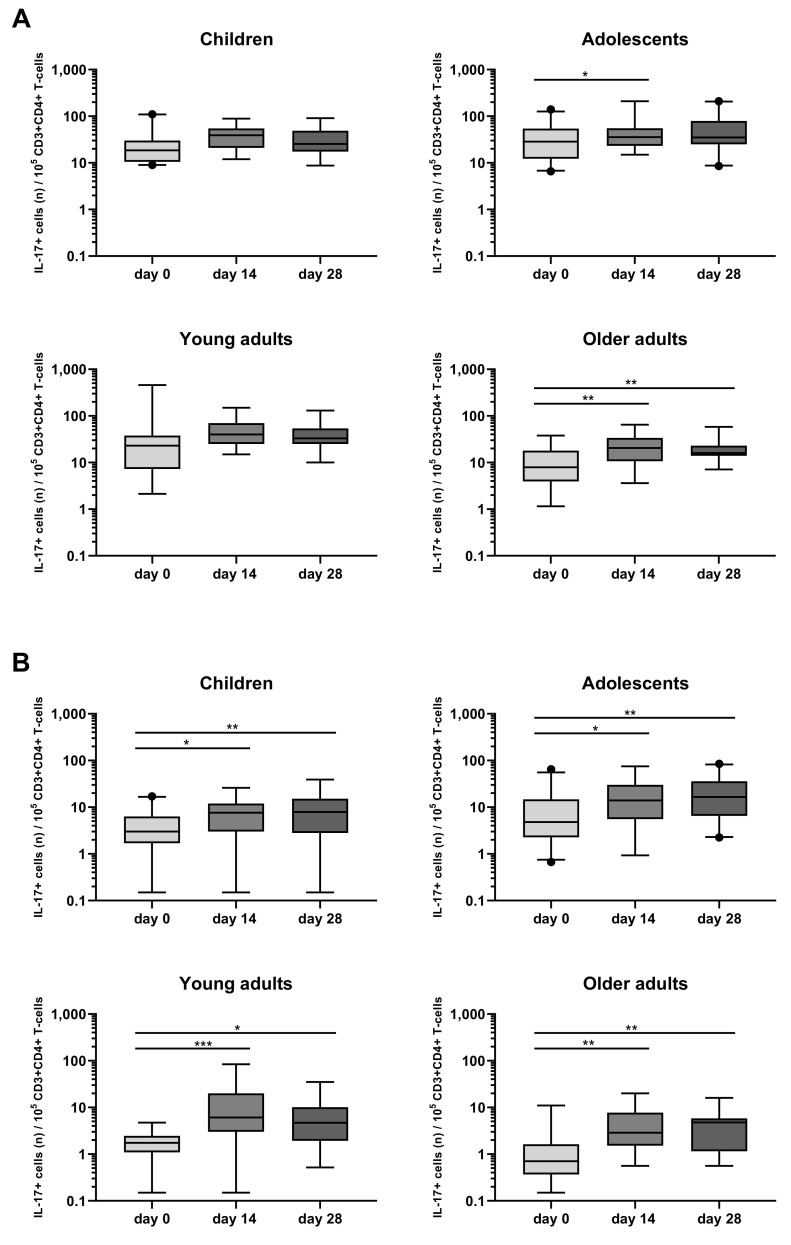
Longitudinal IL-17+ responses in CD4+ T-cells after PT and FHA stimulation in the different age cohorts. Whole blood samples were stimulated with antigens (**A**) PT, and (**B**) FHA, and longitudinal IL-17+ responses were determined after culture by intracellular combined cytokine staining for IL-17A and IL-17F in children, adolescents, young, and older adults, as indicated. Responses are expressed as positive events per 10^5^ CD3+CD4+ T-cells. Boxplots indicate median value, quartile range, and 5–95 percentiles. Circles indicate data points outside the 5-95 percentiles. * *p* < 0.05, ** *p* < 0.01, *** *p* < 0.001.

A rise in Bp specific IL-17+CD4+ T-cells was also observed in the four age cohorts, mostly in response to FHA, with peak rates of IL-17+CD4+ T-cells at days 14 and 28 (Figure 7B, Table S3). In response to PT, a significant rise in the frequency of IL-17+CD4+ T-cells was mostly observed in older adults, both at days 14 and 28. Notably, the frequency of their IL-17+ and IFNγ+CD4+ cells before the booster was very low (Figure 7A, Table S3).

No significant changes in the rate of IL-5/IL-13+CD4+ T-cells after PT and FHA stimulation were detected after the booster vaccination (Figure S2A,B and Table S4). Significant elevations of IL-22+CD4+ T-cells were observed after PT stimulation in adolescents (day 14) and older adults (day 14, day 28) (Figure S3A,B), and after FHA stimulation in children and adolescents.

### 3.6. Primary Vaccination Background Impacts Booster-Induced Bp Specific CD4+ T-Cell Cytokine Profile Later in Life

**Figure 8:**
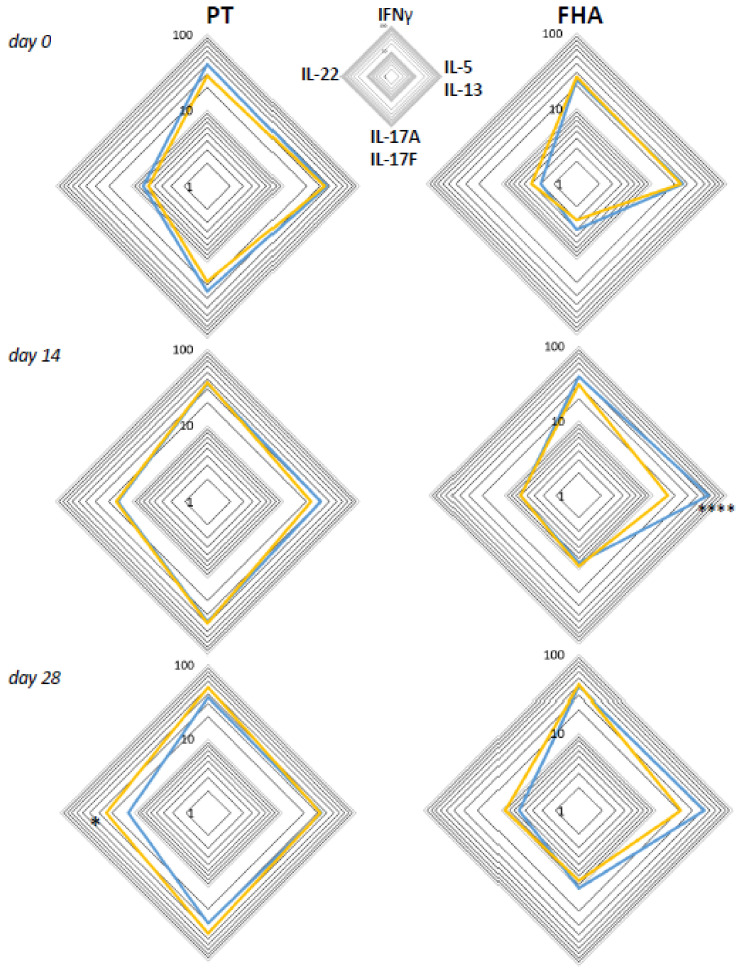
Differences between intracellular cytokine patterns of wP and aP primed cohorts are most apparent in Th2 axes. Geomeans of events positive for intracellular cytokines IFNγ, IL-5/IL-13, IL-17A/F, or IL-22 per 10^5^ CD3+CD4+ T-cells at the four different axes are shown for the subgroup with known aP priming background (children and 46% of adolescents, blue lines) and the subgroup with known wP priming background (54% of adolescents and young adults, yellow lines) after whole blood stimulation with PT (left panels) and FHA (right panels) at days 0, 14, and 28, as indicated. * *p* < 0.05, **** *p* < 0.0001.

Based on literature, early differences in Th lineage cytokine profiles of CD4+ T-cells may still be detectable after booster vaccination later in life [12,13,14]. Therefore, we analyzed the functional profiles of Bp specific CD4+ T-cell responses in children, adolescents, and young adults, with a known primary vaccination background, i.e., either aP (children and part of the adolescents) or wP (other art of adolescents and young adults) vaccination background. Geomean cytokine responses of IFNγ+, IL-5/IL-13+, IL-17+, and IL-22+ events per 10^5^ CD4+ T-cells from the wP versus aP primed subgroups were plotted in radar charts for timepoints days 0, 14, and 28 (Figure 8). The aP primed subgroup was characterized by significantly higher IL-5/IL-13+ events per 10^5^ CD4+ T-cells after stimulation with FHA at day 14, whereas the wP primed subgroup showed significantly higher IL-22+ events per 10^5^ CD4+ T-cells after PT stimulation at day 28. 

### 3.7. Influence of the Primary Vaccination Background on the Cytokine Responses in Supernatants

**Figure 9:**
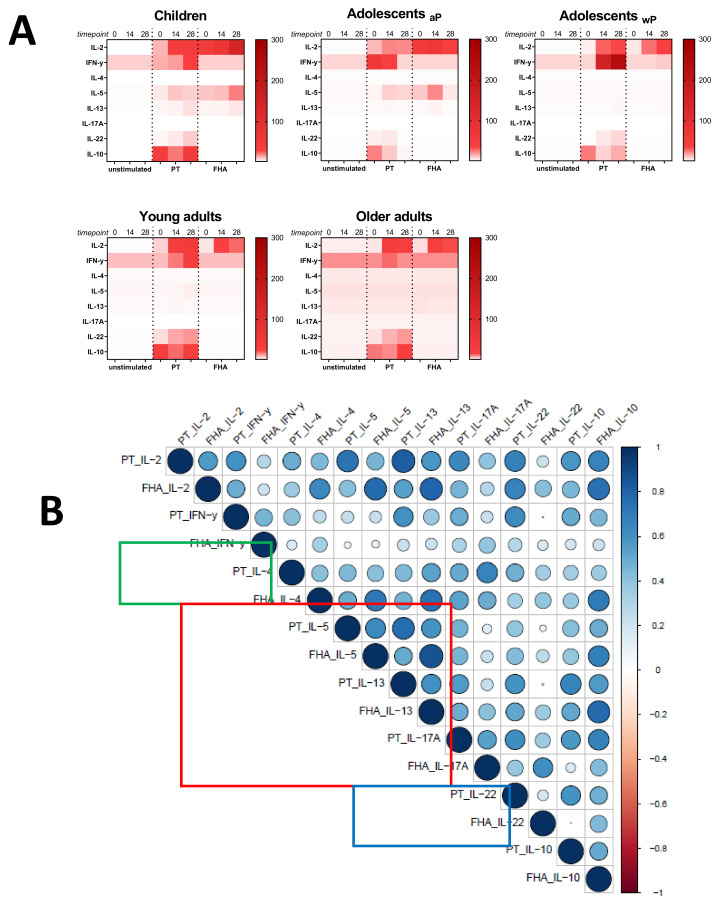

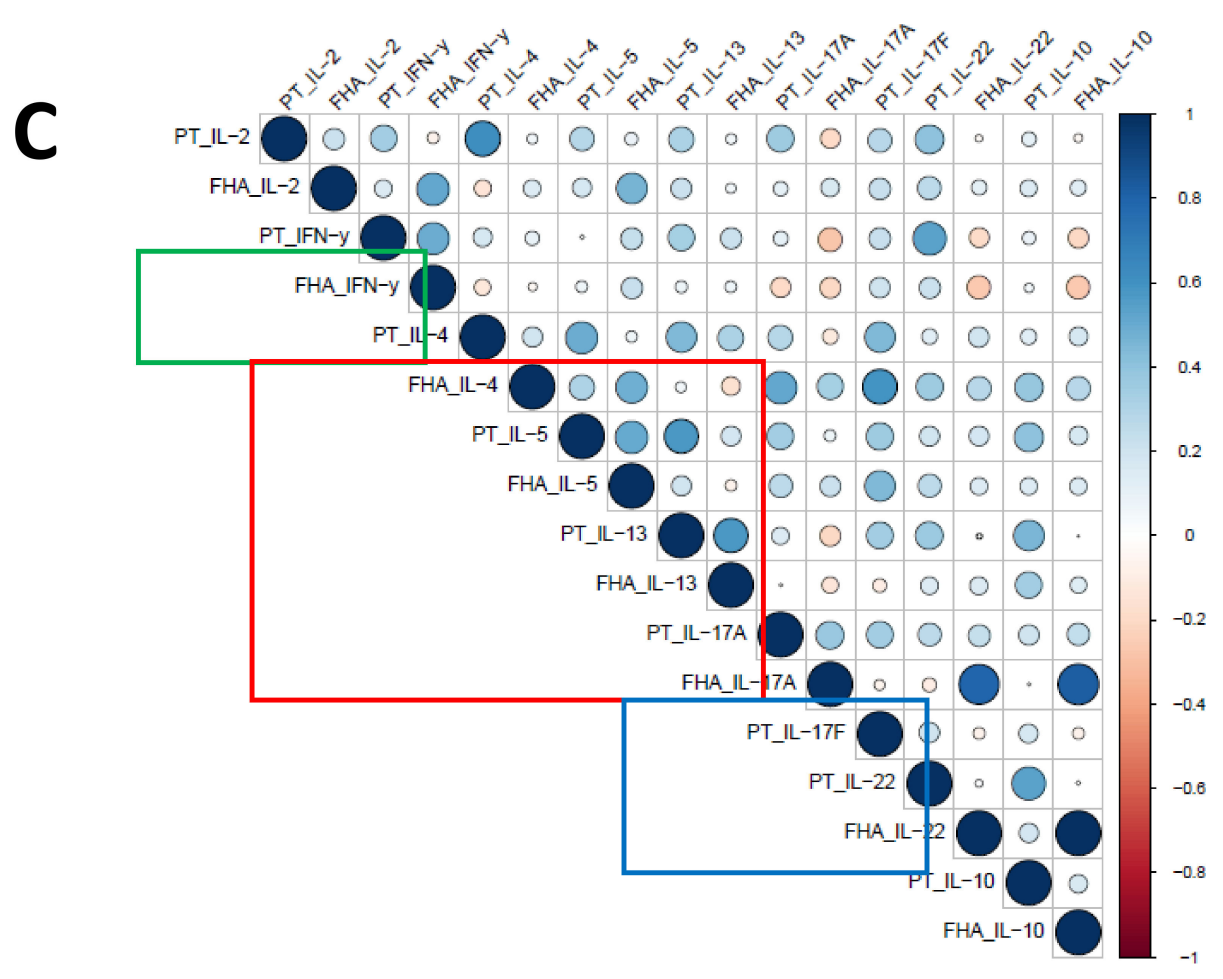
Cytokine levels in supernatants after short incubation with Bp antigens. Diluted whole blood samples from all age cohorts were stimulated for 19 h with PT or FHA as indicated. (**A**) Heatmaps show median concentrations of selected Th lineage associated cytokines (left axis) in [pg/mL] as indicated by the color intensity (right gradient scales) at timepoint days 0, 14, and 28 (indicated above heatmaps) in unstimulated condition (first cluster of three columns), after stimulation with PT (second cluster), and after stimulation with FHA (third cluster), per age cohort as indicated. (**B**) Spearman correlations of z-transformed cytokines concentrations after background subtraction at day 28 of the subgroup of aP primed children and adolescents. Parameters PT_IL-17F, FHA_IL-17F, FHA_IL-21 were excluded from analyses due to absence of variation after z-transformation. (**C**) Similar as (B), for the subgroup of wP primed adolescents and young adults. Parameters FHA_IL-17F and FH_IL-21 were excluded from analyses. For (B) and (C), the size of the circles indicates the strength of the correlation, the blue and red colors indicate a positive and negative correlation, respectively (see gradient scale). Boxes highlight correlations for IFNγ (green), IL-4, IL-5, and IL-13 (red), and IL-17A and/or IL-17F (blue).

**Figure 10:**
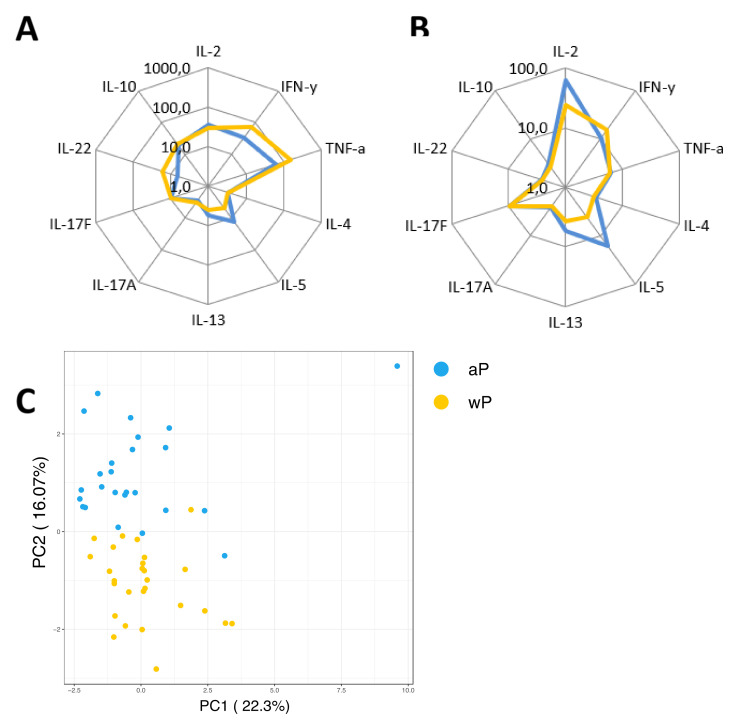
Primary vaccine-dependent patterns of cytokine responses in the supernatant. Diluted whole blood samples were stimulated for 19 h with PT or FHA. (**A**) Spider graph showing geomean concentrations of measured analytes in pg/mL in supernatants, as indicated, for day 28 samples with an aP priming background (children and 46% of adolescents, blue lines) and the subgroup with known wP priming background (54% of adolescents and young adults, yellow lines) after PT stimulation, or (**B**) after FHA stimulation. (**C**) Supernatant based antigen-cytokine measures that can separate the two vaccination groups (aP and wP) were chosen using elastic net. A total of 1000 iterations of elastic net models were generated to separate the subjects on vaccination background and antigen-cytokine pairs that had non-zero coefficient in at least 620 of the 1000 iterations (in the top quartile of four quartiles) were chosen to visualize the separation using a principle component analysis (PCA). The second axis of the PCA, which has high loadings of Th2 and Th1/Th17 cytokines, separates the vaccination backgrounds well.

As a secondary readout, cytokine release in the supernatants of the cultured samples from all age cohorts was also determined. A variety of cytokines covering all Th T-cell lineages was included in the analysis and geomean cytokine levels after PT or FHA stimulation and per timepoint were evaluated in relation to the cohort and primary vaccination background. As expected because of the short incubation and protein arrest within this time frame, the measured concentrations for the cytokines were relatively low (<300 pg/mL). Nevertheless, elevations of early cytokines such as IL-2, IFNγ, and IL-10 were generally detected after antigenic stimulation in response to booster vaccination (Figure 9A). Higher levels of the Th2 related cytokine IL-5 were found in responses of aP primed children and adolescents compared to wP primed adolescents and young adults or the older adults. Next, we explored whether patterns detected in the cell-based readout after stratification by vaccination subgroup were also found in cytokine profiles in the supernatants. Parallel production of individual Th2 related cytokines to PT and FHA stimulations strongly correlated in the combined aP primed subgroup (Figure 9B, red square) but less in the combined wP primed subgroup (Figure 9C, red square). This was also found for IFNγ (green square) and IL-17 (blue square) production. Furthermore, when all Th lineage cytokines were plotted together, both after PT (Figure 10A) and FHA stimulation (Figure 10B) higher geomean IL-5 cytokine level and lower IFNγ and TNF levels were found in day 28 samples in the aP primed subgroup compared to the wP primed subgroup. Ultimately, to minimize variation in age, we aimed to compare adolescents with aP or wP vaccination background regarding their overall cytokine response evaluated. Apart from the statistical analysis of cytokines that are specific to certain primary vaccination backgrounds, we probed if these associations were strong enough to predict the vaccination background of a subject based on all included cytokine responses. We used an elastic net [38] and supernatant data from PT, FHA, and Bp lysate stimulations subtracted with unstimulated controls, to identify which antigen-cytokine pairs can separate the aP primed individuals from wP primed individuals. All 1000 iterations returned an ROC curve (AUC) greater than 0.93. The antigen-cytokine pairs that had a non-zero coefficient in the top quartile of the iterations were chosen as predictors. Using the model, we selected BPL_IL-5, BPL_TNF-a, BPL_IL-22, FHA_IL-5, FHA_TNF-a, FHA_IL-2, BPL_IL-13, FHA_IFN-y, BPL_IL-10, and PT_IL-22 as the stimulation-cytokine pairs that predict the vaccination background (Figure S4A). Indeed, a principal component analysis (PCA) based on those cytokines separates the vaccination backgrounds reasonably well on the second PC (Figure 10C) which can be interpreted as a Th2-Th1/Th17 axis (Figure S4).

## 4. Discussion

The control of pertussis requires the development of next generation vaccines that induce durable protective immunity, including Th1/Th17 lineage memory CD4+ T-cells. Sensitive, specific and qualitative assessment of human CD4+ T-cell immunity to Bp in clinical studies is therefore needed but remains a challenge, especially when limited blood volumes are available such as from infants and children. Here, we applied a novel whole blood assay to determine Th lineage differentiation of *B. pertussis* specific CD4+ T-cells in small volumes of whole blood in a clinical pertussis booster study in various age cohorts. The assay was developed in the framework of the PERISCOPE project [24] and included 19 h stimulation of diluted whole blood samples with various Bp antigens and control conditions, and had as a primary readout flow cytometry-based interrogation of CD4+ T-cells for expression of intracellular cytokines or the activation marker CD154. We show, for the first time in the context of pertussis, that using a whole blood assay it is possible to detect Bp specific CD4+ T-cells of all functional Th lineages at low frequencies. In response to the antigens PT or FHA, median percentages of CD4+ T-cells positive for cytokines at any timepoint in any age cohort typically ranged between 0.04%–0.13%, 0.02%–0.07%, 0.01%–0.11%, and 0.02%–0.11% for IFNγ, IL-5/IL-13, IL-17, and IL-22, respectively. The small differences between frequencies of cytokine producing CD4+ T-cells could reflect true differences in abundance of Th subsets, but we cannot exclude that differences in kinetics of production of Th-lineage cytokines could play a role as well. The overall low frequencies of CD4+ T-cells producing cytokines to Bp antigens correspond well to the low frequencies reported for CD4+ T-cells to other pathogen-derived or vaccine antigens [39,40,41]. Our cell-based readout of CD154 expression in general identified potentially larger median populations of specific CD4+ T-cells (range 0.014%–0.267%) and further confirmed that of the Bp antigens used, the vaccine antigens PT and FHA were, not surprisingly, best suitable to recall specific CD4+ memory T-cells *in vitro* and monitoring their expansion in time after an aP booster vaccination. Although both Bp lysate and Bp peptide pool also contain PT and FHA, either as whole antigens or as peptide epitopes, in the current assay conditions these antigen preparations were associated with background activation or too weak stimulatory capacity, respectively. Of note, the yield of CD4+ T-cells in our cell-based assay using 400 µL whole blood samples per stimulation condition was high, around 40%–50% (despite intense processing of samples), based on absolute cell counting before and acquired absolute cell counts after culture of the 400 µL. Despite significant variation in CD4+ T-cell counts per volume of whole blood as well as in yield after processing, with age, a median of 173,000–193,000 events could be acquired in the CD3+CD4+ T-cells gate for all age cohorts. Nevertheless, to be able to detect a reliable substantial number of rare events in the respective cytokine gates, the sample volume per stimulation conditions could further be optimized e.g., by minimizing the number of antigenic stimulations per sample.

The whole blood assay revealed various biologically meaningful patterns of Bp specific CD4+ T-cell immunity in participants 7–70 years of age. First, we found significantly increased CD154+ and Th1 and Th17 lineage CD4+ T-cell responses during longitudinal follow up of participants after receiving an aP booster vaccination. There was however substantial variation between participants and cohorts in the kinetics of the longitudinal responses, which could be influenced by several factors. It is known that variation in immune responses can be based on underlying circadian rhythms [42,43]. As most participants were sampled around the same time of the day for all timepoints, this likely does not contribute to variation. The cohort of 60–70-year-olds (older adults) showed in general less significant booster responses over time. In this cohort immunosenescence could play a role, possibly affecting in vivo and in vitro CD4+T-cell responsiveness or kinetics (reviewed in [44,45]). Additionally, these older adults may have encountered more natural exposures to circulating *B. pertussis*, possibly affecting their pre-existing immunity, cellular and humoral, and the boostability by a booster vaccine. Throughout all age cohorts, IL-5/IL-13 intracellular responses at day 0 were observed and remained relatively flat over time. This could be interpreted as sustained Th2 memory responsiveness and/or poor boostability of this immune response. Absence of Th2 boostability corroborates with other studies having shown that memory immune responses in aP primed vaccines, skewed towards the Th2 lineage, were poorly boostable [10,13,46].

Second, with this assay it appears possible to predict primary vaccination background based on the balance of detected Th lineages. This trend was weak when using the normalized data from the intracellular cytokine staining. Nevertheless, elastic net model based on antigen-cytokine pairs from the supernatant readouts was able to identify vaccination background with an ROC of at least 0.93. Visualization of the samples using principal component analysis (PCA) showed separation of vaccination background on the second principal component. It is to be noted that the first component correlates with stimulant antigen the second axis correlates with Th2-Th1/Th17 cytokine signal.

Interestingly, in this secondary supernatant readout, we found significant cytokine levels even though the incubation time of the assay was only 19 h and arrest of protein transport was applied to capture the cytokines inside the cell. Yet, the levels of IL-5, IL-13, and IL-17 in the supernatant were just above detection level, confirming that for proper detection in the supernatant, a longer stimulation would be required. A disadvantage of supernatant analyses is that it is not possible to distinguish which immune cells contributed to the production of detected cytokines. We observed varying levels of IL-6, indicating the contribution of innate cells to the whole blood response within the 19 h time frame. However, since certain cytokine trends found in the flow cytometry-based CD4+ T-cell readout were reflected in the supernatant readout, we propose that the activation of innate cells in the whole blood assay did not disturb the readout of Th lineage cytokines in the supernatants. In fact, we propose that innate cells in the assay could contribute to a pro-inflammatory milieu that may enhance the activation and detection of specifically responding CD4+T-cells. Likewise, the presence of autologous serum components in the whole blood assay, including Bp specific antibodies, may add relevant context to the detection of CD4+T-cells during steady state immunity or recall responses. Currently, evaluation of serum antibodies in the booster study is ongoing, enabling assessment of correlation between humoral and cell-mediated immune readouts.

Our results show that Bp specific CD4+T-cells can be detected based on expression of activation marker CD154 and intracellular cytokine detection using a novel assay. For further optimization of the whole blood Bp specific CD4+T-cell platform, there are some recommendations. Feasibility of the cell-based format in clinical studies could be enhanced by introducing a freezing step in the protocol, by which cell pellets generated after the real-time culture step can be stored for more efficient batch-wise downstream processing, staining, and data acquisition. A versatile format with supernatant-based readout only could be derived by optimizing cytokine production in the supernatant through a prolonged culture time and leaving out the protein transport inhibition step. Depending on the research question and endpoint involved, we expect that either of such optimized whole blood assays could be valuable in future clinical studies of Bp specific CD4+T-cell immunity.

## 5. Conclusions

We showed for the first time that analysis of Bp specific CD4+ T-cell memory in whole blood reveals vaccine booster responses with hallmarks imprinted by primary vaccination and, if further optimized, provides a promising tool to assess the quantity and quality of CD4+ T-cell responses induced by novel pertussis vaccine candidates.

## Figures and Tables

**Table 1:** Cohort description.

Characteristic	Children 7–10 yrs (*n* = 19)	Adolescents 11–15 yrs (*n* = 24)	Young Adults 20–34 yrs (*n* = 15)	Older Adults 60–70 yrs (*n* = 15)
Sex–no. (%)				
Male	10 (53)	16 (67)	9 (60)	10 (67)
Female	9 (47)	8 (33)	6 (40)	5 (33)
Age–yrs				
Median	8	13,5	28	65
Range	7–9	11–15	21–34	61–69
Primary Vaccination Background				
Acellular Pertussis Vaccination	100%	46%	0%	0%
Whole-Cell Pertussis Vaccination	0%	54%	100%	Unknown proportion
No Vaccination	0%	0%	0%	Unknown proportion

## Supplementary Materials

The following are available online at https://www.mdpi.com/2076-393X/8/2/225/s1, Table S1: Rate of cytokine producing CD3+CD4+ T-cells after various in vitro stimuli, Table S2: Rate of IFNγ producing CD3+CD4+ T-cells after PT and FHA stimuli, Table S3: Rate of IL-17A/IL-17F producing CD3+CD4+ T-cells after PT and FHA stimuli, Table S4: Rate of IL-5/IL-13 producing CD3+CD4+ T-cells after PT and FHA stimuli, Figure S1: Consistent high levels of CD154+ events in CD3+CD4+ T-cells after *Staphylococcus aureus* enterotoxin B (SEB) stimulation over time, Figure S2: Longitudinal IL-5/IL-13+ responses in CD4+ T-cells after PT and FHA stimulation in the different age cohorts, Figure S3: Longitudinal IL-22+ responses in CD4+ T-cells after PT and FHA stimulation in the different age cohorts, Figure S4: Results from 1000 iterations of elastic-net and PCA axis loadings plot.
