# Supplementary material for: Uncovering Distinct Primary Vaccination-Dependent Profiles in Human Bordetella pertussis Specific CD4+ T-Cell Responses Using a Novel Whole Blood Assay
Source: Vaccines (Basel). 2020 May 15;8(2):225. doi: 10.3390/vaccines8020225 (PMC7349943; doi:10.3390/vaccines8020225)
Supplement: Supplementary file 1 [file vaccines-08-00225-s001.pdf]

Supplementary materials:

Table S1. Rate of cytokine producing CD3+CD4+ T-cells after various in vitro stimuli.

Readout	Stimulation	Geomean#	5-95 percentile	p-value*
IFNγ	unstimulated	18	3 – 86	
	PT	64	14 – 490	<0.0001
	FHA	29	9 – 119	0.0149
	Bp lysate	77	20 – 299	<0.0001
	Peptide pool	23	9 – 60	>0.9999
IL-5/IL-13	unstimulated	17	4 – 84	
	PT	31	8 – 130	<0.0001
	FHA	25	4 – 140	0.0015
	Bp lysate	296	7 – 167	0.0006
	Peptide pool	21	4 – 105	0.8145
IL-17A/IL-17F	unstimulated	1	1 – 10	
	PT	26	6 – 110	<0.0001
	FHA	5	1 – 39	<0.0001
	Bp lysate	78	15 – 299	<0.0001
	Peptide pool	3	1 – 21	0.2142
IL-22	unstimulated	4	1 – 41	
	PT	12	1 – 83	<0.0001
	FHA	4	1 – 31	0.8370
	Bp lysate	73	11 – 318	<0.0001
	Peptide pool	3	1 – 24	0.0049

median number of cytokine positive events per 10^5 CD3+CD4+ T-cells for all age cohorts and time points combined. * *p* value for comparisons of stimulated conditions to unstimulated controls.

Table S2. Rate of IFNγ producing CD3+CD4+ T-cells after PT and FHA stimuli.

Cohort	Day	PT		FHA	
		Median#	p-value*	Median#	p-value*
**Children**	0	49		26	
	14	205	0.0002	33	0.0019
	28	140	0.0009	32	0.4729
Adolescents	0	40		26	
	14	220	<0.0001	19	0.0077
	28	190	<0.0001	16	0.0027
Young adults	0	22		26	
	14	200	0.0002	25	0.5898
	28	200	0.0002	29	0.5083
Older adults	0	22		15	
	14	130	0.0002	27	0.1677
	28	89	0.0003	20	0.7929

# number of IFNγ positive events per 10^5 CD3+CD4+ T-cells per age cohort at the different timepoint.
* *p* value for comparisons of response levels at day 14 and day 28 to levels on day 0 .

Table S3. Rate of IL-17A/IL-17F producing CD3+CD4+ T-cells after PT and FHA stimuli.

**Cohort**	Day	PT		FHA	
		Median#	p-value*	Median#	p-value*
**Children**	0	19		3	
	14	29	0.1294	8	0.0245
	28	26	0.1677	8	0.0023
Adolescents	0	29		5	
	14	36	0.0215	14	0.0432
	28	35	0.0853	17	0.0081
Young adults	0	23		2	
	14	40	0.0946	6	0.0003
	28	33	0.1040	5	0.0107
Older adults	0	8		1	
	14	21	0.0034	2	0.0061
	28	16	0.0031	1	0.0034

number of IL-17A/IL-17F positive events per 10^5 CD3+CD4+ T-cells per age cohort at the different timepoints. * *p* value for comparisons of response levels at day 14 and day 28 to levels on day 0 .

Table S4. Rate of IL-5/IL-13 producing CD3+CD4+ T-cells after PT and FHA stimuli.

Cohort	Day	PT		FHA	
		Median#	p-value*	Median#	p-value*
Children	0	55		38	
	14	25	0.2744	4	0.0809
	28	28	0.2166	50	0.1099
Adolescents	0	33		22	
	14	33	0.7379	31	0.0569
	28	39	0.8404	35	0.0675
Young adults	0	34		26	
	14	31	0.1556	10	0.1205
	28	32	0.6698	29	0.4356
Older adults	0	24		15	
	14	26	0.3657	15	0.9515
	28	24	0.3303	17	0.8469

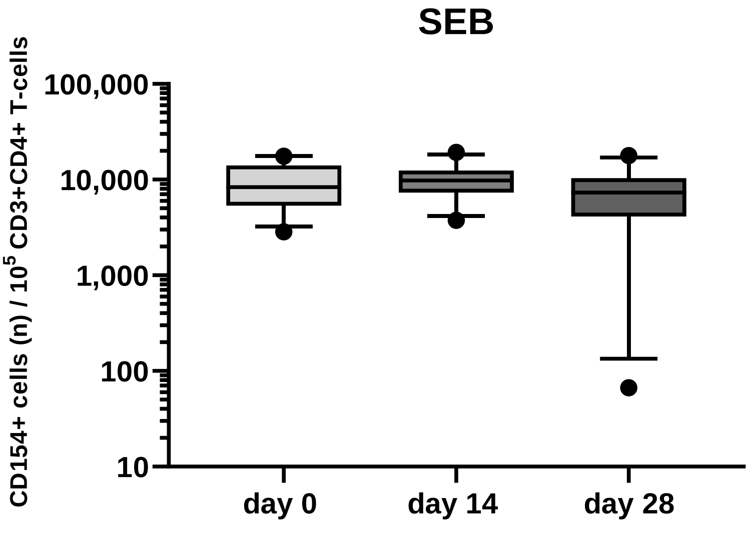

Figure S1. Consistent high levels of CD154+ events in CD3+CD4+ T-cells after SEB stimulation over time. CD154+ events per 10^5 CD3+CD4+ T-cells were determined in whole blood samples from all 4 age cohorts, at all timepoints and after stimulation with SEB [1 μg/mL]. Boxplots indicate median value, quartile range and 5–95 percentiles. Circles indicate data points outside the 5-95 percentiles.

A

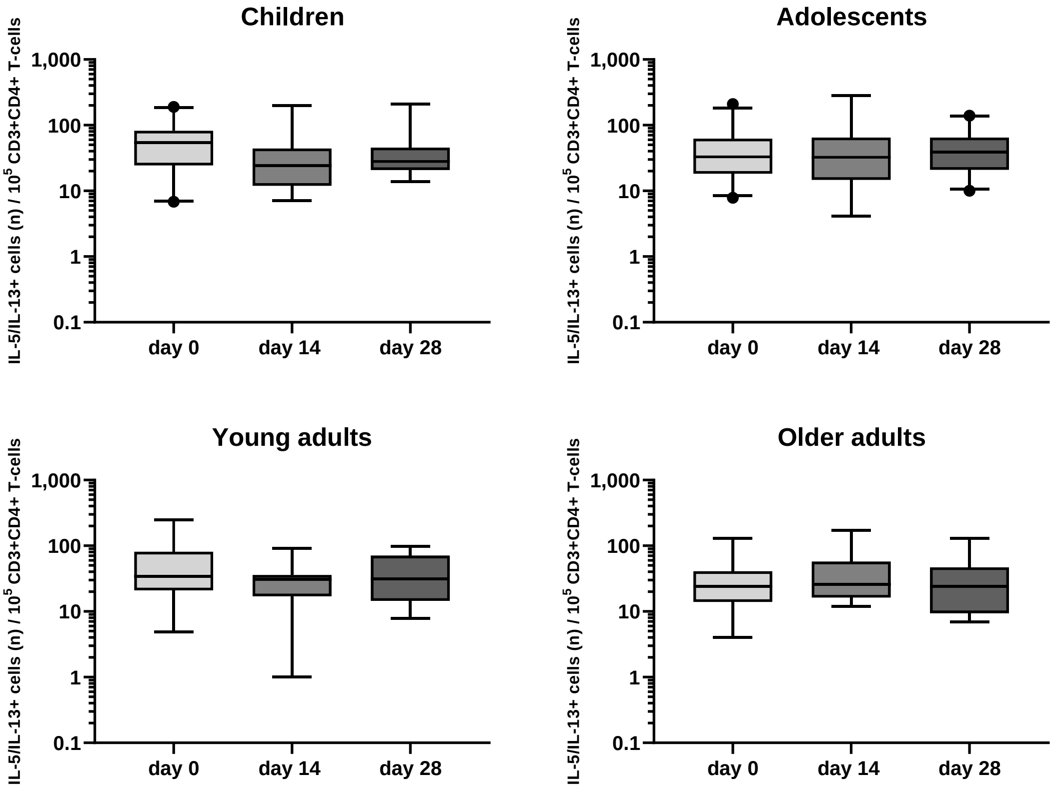
Children
Adolescents
Young adults
Older adults
IL-5/IL-13+ cells (n) / 10^5 CD3+CD4+ T-cells
1,000
100
10
1
0.1
day 0
day 14
day 28

B

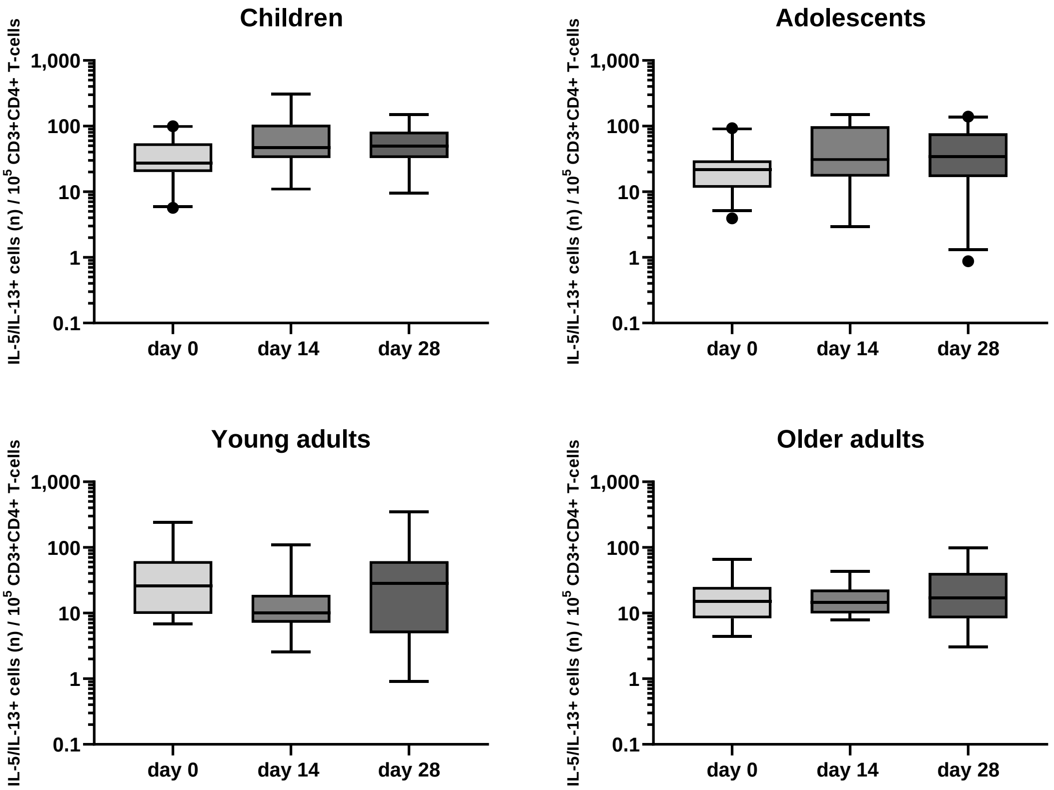
Children
Adolescents
Young adults
Older adults
IL-5/IL-13+ cells (n) / 10^5 CD3+CD4+ T-cells
1,000
100
10
1
0.1
day 0
day 14
day 28

Figure S2. Longitudinal IL-5/IL-13+ responses in CD4+ T-cells after PT and FHA stimulation in the different age cohorts. Whole blood samples were stimulated with antigens (**A**) PT and (**B**) FHA and longitudinal IL-5/IL-13 responses were determined after culture by intracellular cytokine staining in cohorts, as indicated. Responses are expressed as cytokine positive events per 10^5 CD3+CD4+ T-cells. Boxplots indicate median value, quartile range and 5–95 percentiles. Circles indicate data points outside the 5-95 percentiles.

A

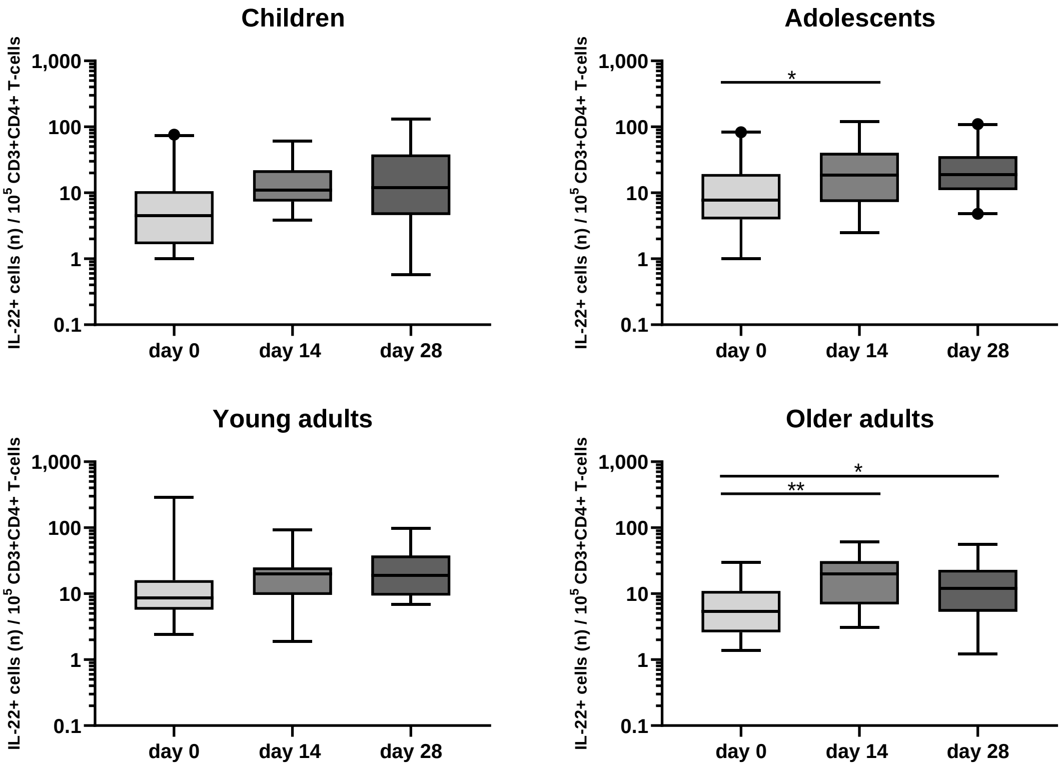
Children
Adolescents
Young adults
Older adults
IL-22+ cells (n) / 10^5 CD3+CD4+ T-cells
1,000
100
10
1
0.1
day 0
day 14
day 28
*
**

B

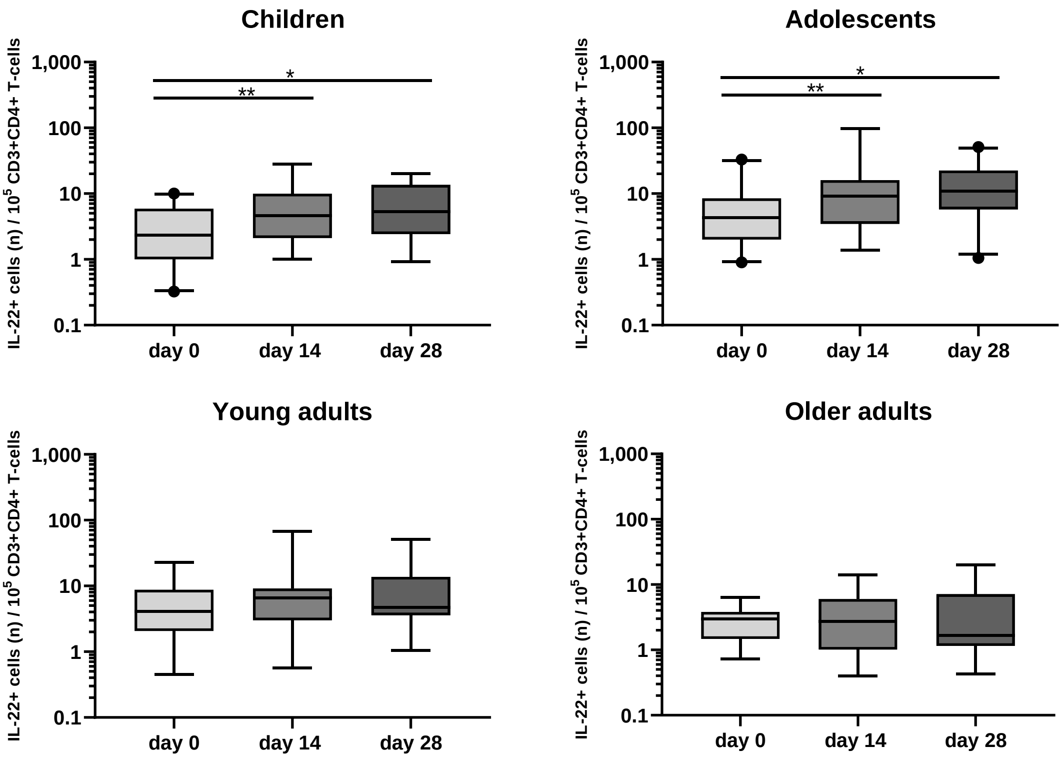
Children
Adolescents
Young adults
Older adults
IL-22+ cells (n) / 10^5 CD3+CD4+ T-cells
1,000
100
10
1
0.1
day 0
day 14
day 28
*
**

Figure S3. Longitudinal IL-22+ responses in CD4+ T-cells after PT and FHA stimulation in the different age cohorts. Whole blood samples were stimulated with antigens (**A**) PT and (**B**) FHA and longitudinal IL-22 responses were determined after culture by intracellular cytokine staining in cohorts, as indicated. Responses are expressed as cytokine positive events per 10^5 CD3+CD4+ T-cells. Boxplots indicate median value, quartile range and 5-95 percentiles. Circles indicate data points outside the 5-95 percentiles. * $p < 0.05$, ** $p < 0.01$.

A

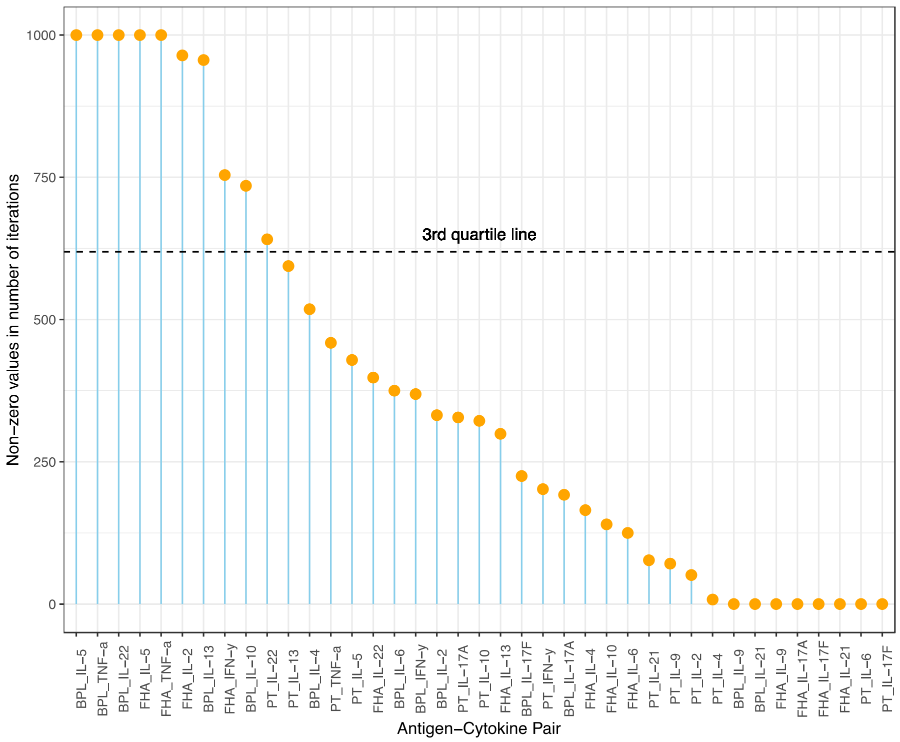

B

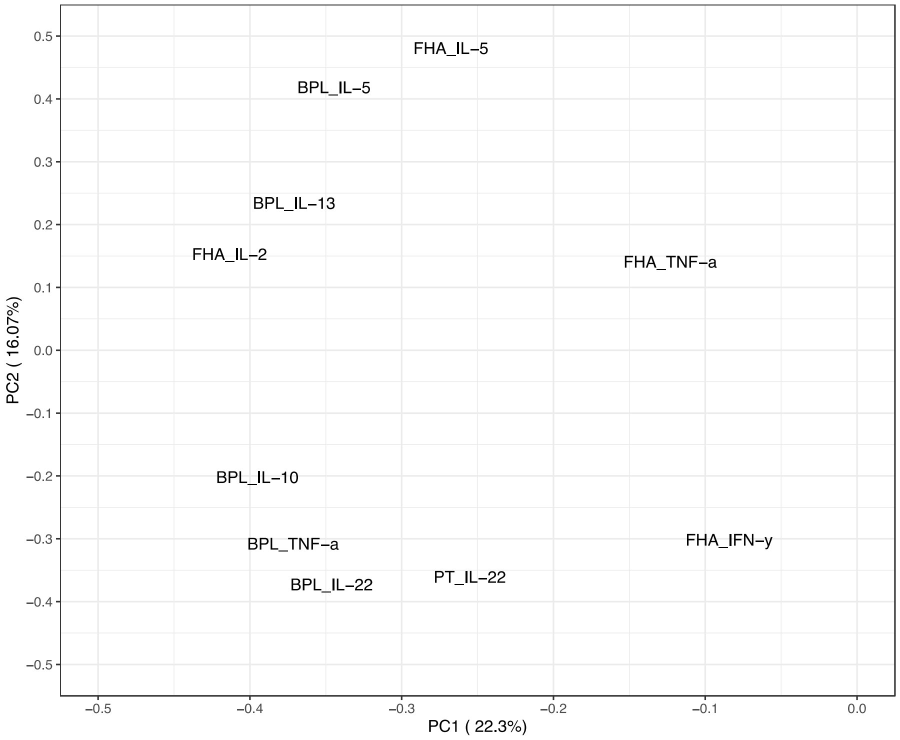

Figure S4. Results from 1000 iterations of elastic net and PCA axis loadings plot. (**A**) Counts of antigen-cytokine pairs that had non-zero coefficient in each iteration after 1000 iterations of elastic net regression models. The antigen-cytokine pair that had coefficients more than the 3rd quartile of the count values were chosen for PCA analysis. (**B**) PCA loadings plot showing the loadings on each antigen-cytokine pair for the first two PCs. BPL, Bp lysate.
